# Intensive versus conventional phototherapy for neonatal hyperbilirubinemia: a systematic review and meta-analysis of RCTs and cohort studies

**DOI:** 10.3389/fmed.2026.1862217

**Published:** 2026-06-09

**Authors:** Yuan Qiao, Min Song, Guoying Lu, Fan Liu, Hongling Cao, Meifen Yang

**Affiliations:** Department of Neonatology, The Fourth Affiliated Hospital of Dali University, Dali, Yunnan, China

**Keywords:** neonatal hyperbilirubinemia, intensive phototherapy, conventional phototherapy, total serum bilirubin, light irradiance

## Abstract

**Background:**

The clinical efficacy of intensive phototherapy for neonatal hyperbilirubinemia remains inconsistently reported across studies.

**Method:**

The meta-analysis was conducted in accordance with PRISMA 2020 and MOOSE guidelines. We searched Web of Science, PubMed, Embase, and ScienceDirect for eligible comparative studies from the inception of the databases up to March, 2026. The primary outcome was the reduction in total serum bilirubin (TSB) levels from baseline to the completion of phototherapy, and the duration of phototherapy was defined as the secondary outcome. The pooled results were synthesized via random-effect model. Influential publication was determined by performing sensitivity analysis. In addition, the potential sources of heterogeneity were examined by using subgroup analyses. Publication bias was assessed using the funnel plot, Begg’s and Egger’s tests.

**Result:**

The 12 included studies (8 RCTs and 4 cohort studies) published between 1995 and 2026, enrolling 1,144 neonates with mean gestational age ranging from 30 to 39 weeks and baseline TSB levels from 172 to 390 μmol/L. Intensive phototherapy achieved significantly greater TSB reductions compared to conventional phototherapy within the same timeframe (MD = −21.87, 95% CI = −29.69 to −14.05, *p* < 0.001, I^2^ = 83.9%). Subgroup analysis showed that the gestational age (*p* = 0.003) and baseline TSB level (*p* = 0.005) were potential source of heterogeneity, but study design was not (*p* = 0.106). In addition, the treatment duration from the intensive phototherapy group were shorter than the conventional group (MD = −20.23 h, 95% CI = −38.91 to −1.54, *p* = 0.034, I^2^ = 94.7%). No influential publications and significant publication bias were detected across the 12 studies.

**Conclusion:**

Phototherapy has been established as the cornerstone of clinical management for neonatal hyperbilirubinemia. Intensive phototherapy demonstrates superior clinical efficacy in reducing TSB levels and treatment duration than conventional phototherapy, especially in preterm neonates with severe hyperbilirubinemia.

**Systematic review registration:**

https://www.crd.york.ac.uk/PROSPERO/view/CRD420261324708, Identifier CRD420261324708.

## 1 Introduction

Neonatal jaundice, clinically defined as hyperbilirubinemia, represents a ubiquitous condition in neonatology and constitutes the primary etiology for hospital readmissions worldwide (1). Clinical manifestations of jaundice typically become apparent when total serum bilirubin (TSB) concentrations surpass the threshold of 7 mg/dL. Epidemiologically, this condition exhibits a prevalence ranging from 25 to 50% among term infants, whereas a significantly higher incidence is characteristic of the preterm population (2). The global burden of severe neonatal hyperbilirubinemia demonstrates marked regional heterogeneity. Prevalence is highest in the African region (667.8 per 10,000 livebirths), followed by the South-East Asia region (251.3 per 10,000 livebirths), while rates are substantially lower in the Americas region (3.7 per 10,000 livebirths) (3). From a pathophysiological perspective, neonatal jaundice is stratified into two distinct phenotypes: indirect (unconjugated) and direct (conjugated) hyperbilirubinemia. A critical clinical distinction exists regarding their sequelae: whereas direct hyperbilirubinemia is rarely associated with neurological impairment, indirect hyperbilirubinemia exhibits significant neurotoxicity, thereby posing a substantial risk of detrimental central nervous system injury (2). Given the profound neurotoxic risks, timely and effective therapeutic intervention is paramount to mitigate neurological sequelae.

Phototherapy represents the cornerstone of clinical management for neonatal jaundice, widely adopted due to its established safety profile and non-invasive nature. In high-income countries, the implementation of phototherapy has been instrumental in virtually eliminating the necessity for exchange transfusion in severe hyperbilirubinemia, concurrently precipitating a marked decline in the incidence of acute bilirubin encephalopathy (4). The therapeutic management of unconjugated hyperbilirubinemia is primarily predicated on phototherapy, a modality that induces the photo-isomerization of bilirubin into water-soluble configurational isomers and lumirubin, thereby facilitating biliary and urinary excretion independent of hepatic conjugation (5). The clinical efficacy of phototherapy is governed by specific physicochemical parameters of the light source, such as peak emission wavelength, spectral bandwidth, and light irradiance, in addition to a multitude of clinical variables (6). Current guidelines from the American Academy of Pediatrics recommend phototherapy utilizing blue light be administered with an irradiance of <30 μW/cm^2^/nm, a source-to-skin distance of at least 10 cm, and continuous monitoring of the infant’s thermal status (7). Historically, conventional phototherapy has been administered via electrically powered systems, which necessitate a continuous power supply and rigorous monitoring to ensure irradiance levels are maintained within the prescribed therapeutic window (8). While the administration of standard phototherapy was refined, the necessity for accelerated bilirubin clearance to minimize neurotoxicity has driven the adoption of intensive phototherapy regimens.

Conventional phototherapy modalities predominantly utilize fluorescent tubes and halogen spotlights. However, their substantial thermal emission precludes close proximity to the infant, thereby limiting irradiance intensity. The light-emitting diodes (LEDs) have emerged as a superior alternative, characterized by negligible heat generation, which permits safe placement in immediate proximity to the neonate (9). Research also indicates that conventional phototherapy is significantly less efficacious than intensive phototherapy, often necessitating a treatment duration four times longer. Consequently, intensive phototherapy is considered more cost-effective, as the abbreviated treatment period reduces the overall hospital length of stay (10). Despite the established advantages, the clinical efficacy of intensive phototherapy remains inconsistently reported across studies, with a paucity of robust meta-analysis to consolidate these findings. However, current systematic reviews and meta-analyses have largely centered on hardware configurations or care settings, often neglecting the critical variable of light intensity (11, 12). The failure to incorporate the dose–response relationship governed by irradiance may mask the actual therapeutic benefits of intensive phototherapy in these analyses. While randomized controlled trials (RCTs) minimize bias, they often have limited sample sizes and strict inclusion criteria. Conversely, observational studies reflect real-world clinical scenarios and provide data on larger, more diverse populations. Therefore, we included both study designs in this meta-analysis to ensure the most robust and comprehensive evidence.

Here, we conducted a systematic review and meta-analysis to clarify the efficacy of intensive phototherapy. Our research question was explicitly defined using the PICO framework: to evaluate whether intensive phototherapy (I), compared with conventional phototherapy (C), leads to a greater reduction in serum bilirubin and shorter treatment duration (O) in neonates with hyperbilirubinemia (P).

## 2 Methods

The study was in line with Preferred Reporting Items for Systematic Reviews and Meta-Analysis (PRISMA) and MOOSE Guidelines (13, 14). The protocol for this systematic review and meta-analysis was registered on PROSPERO (ID: CRD420261324708).

### 2.1 Eligibility criteria

This meta-analysis includes studies comparing the efficacy of intensive (light irradiance ≥30 μW/cm^2^/nm) versus conventional (light irradiance <30 μW/cm^2^/nm) phototherapy in neonates (≤14 days) with hyperbilirubinemia (TSB > 170.0 μmol/L). The primary outcome was defined as the reduction in TSB levels (μmol/L) from baseline to the completion of phototherapy. The duration of phototherapy (hour) was defined as the secondary outcome. Studies that meet one of the following criteria were excluded: (1) laboratory-based research; (2) non-English language; or (3) full text unavailable. Publication date, study design, sample size and clinical setting were not considered as the basis for exclusion.

### 2.2 Search strategy

The literature search was conducted in accordance with the PICO framework, focusing on neonates with hyperbilirubinemia (P), intensive versus conventional phototherapy (I), and clinical efficacy (O). The Web of Science, PubMed, ScienceDirect and Embase databases were screened from the inception of the databases up to March, 2026. Besides, a manual search of reference lists from relevant reviews and meta-analyses was performed to identify further eligible studies. The search strategy utilized the following query: (Neonatal hyperbilirubinemia or Newborn jaundice or Neonat* jaundice) and (Phototherapy or Phototherap* or Light therapy or Blue light).

### 2.3 Study selection

After the retrieval of studies from the databases, records were deduplicated using EndNote software. Two reviewers independently screened the titles and abstracts for relevance to the study objectives. Finally, the full texts of potentially eligible studies were assessed against the inclusion and exclusion criteria. Any discrepancies between the reviewers were resolved through discussion or by consulting a third reviewer. Cohen’s kappa (*κ*) was used to measure the agreement on inclusion and exclusion decisions.

### 2.4 Quality assessment

Two reviewers independently assessed the risk of bias for the included randomized controlled trials (RCTs) using the Cochrane Risk of Bias Tool version 2 (RoB2) (15), and evaluated the methodological quality of the cohort studies with the Newcastle-Ottawa Scale (NOS) checklist (16). Any discrepancies were resolved by consulting a third reviewer. The level of agreement for the risk of bias assessment was measured using the Cohen’s *κ*.

### 2.5 Data extraction and synthesis

Data extraction was performed independently by two reviewers, with discrepancies resolved through discussion or adjudication by a third reviewer. There was almost perfect agreement between the reviewers regarding the extracted data (Cohen’s *κ* = 0.95). A standardized data collection form was used to extract the following variables: first author, publication year, country of origin, study design, sample size, gestational age, mean initial total serum bilirubin (TSB) levels, device type, and duration of phototherapy. The reduction in TSB levels (μmol/L) and duration of phototherapy (hour) were expressed as mean difference (MD) with 95% confidence interval (95% CI). Statistical heterogeneity was evaluated using the I^2^ statistic and Cochran’s Q test, where the values of 25, 50, and 75% were interpreted as low, moderate, and high heterogeneity, respectively. A random-effects model was employed when moderate to high heterogeneity was detected; otherwise, a fixed-effects model was applied. Influential publication was determined by performing sensitivity analysis. In addition, potential source of heterogeneity was identified by subgroup analysis. Publication bias was assessed by using the funnel plot, Begg’s and Egger’s tests.

### 2.6 Statistical analysis

Statistical analyses were performed using Stata 16 software. The “metan,” “metaninf,” “metafunnel,” and “metabias” commands were utilized to conduct the pooled analyses, sensitivity analyses, and publication bias assessments, respectively. All statistical tests were two-tailed, with a *p*-value <0.05 considered statistically significant.

## 3 Results

This study presents a comprehensive meta-analysis comparing the clinical efficacy of Intensive versus conventional phototherapy for neonatal hyperbilirubinemia, while also investigating potential factors associated with treatment outcomes.

### 3.1 Study selection

**Figure 1:**
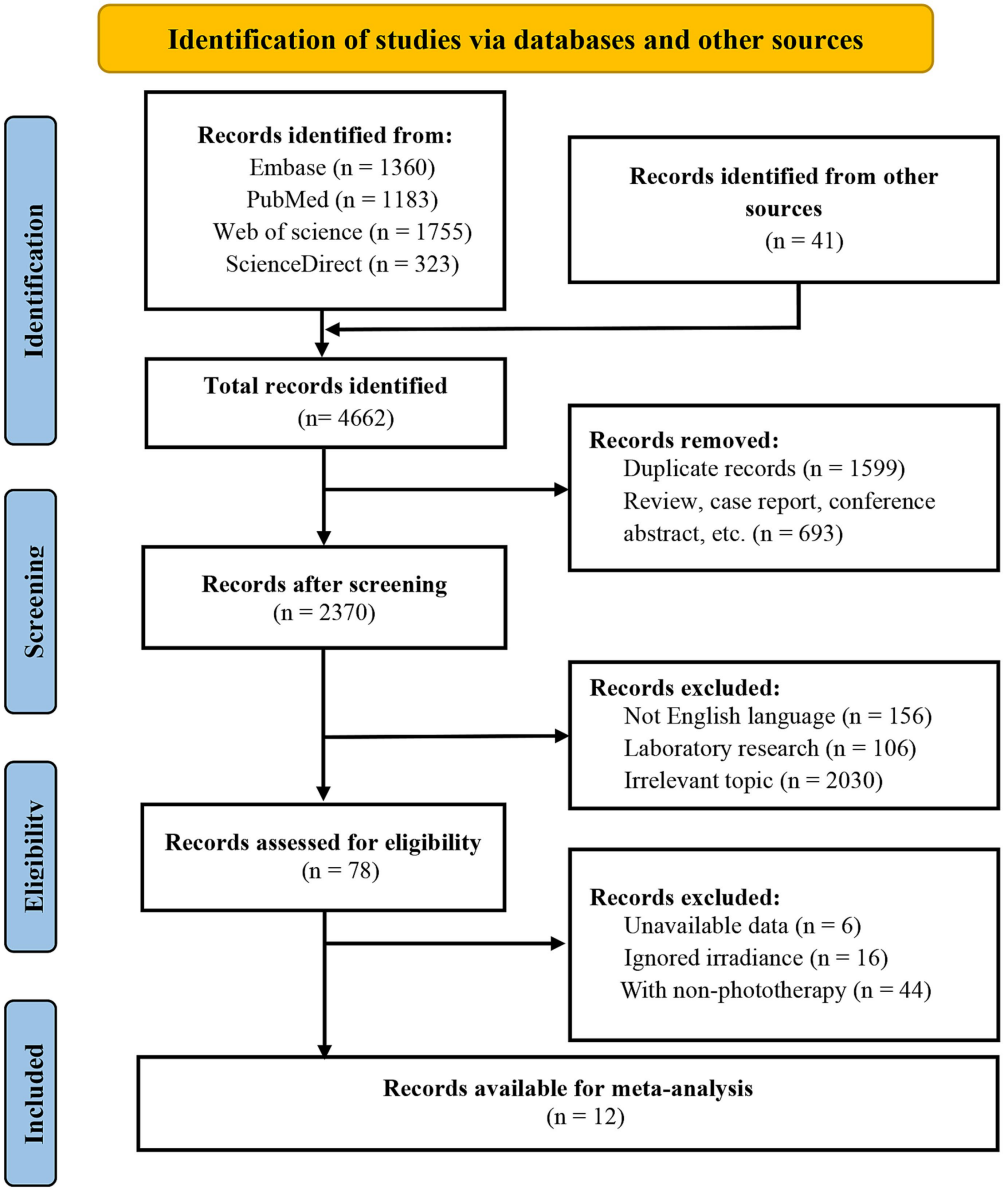
PRISMA flowchart of 12 included studies.

The study selection process is presented in Figure 1. An initial search yielded 4,621 records from electronic databases, supplemented by 41 additional studies identified through manual screening of reference lists. After removing duplicates and excluding irrelevant studies based on title and abstract, 78 articles remained for full-text eligibility assessment. Of these, 66 were excluded due to unavailable data (*n* = 6), lack of categorization by irradiance intensity (*n* = 16), or comparison with non-phototherapy interventions (*n* = 44). Ultimately, 12 studies met the inclusion criteria were included in the final analysis (2, 17–27). The agreement between reviewers during the study selection process was substantial (Cohen’s *κ* = 0.81).

### 3.2 Study characteristics and quality assessment

The 12 included studies (8 RCTs and 4 cohort studies) were conducted between 1995 and 2026. These studies enrolled a total of 1,144 patients, with sample size ranging from 31 to 200 participants. The mean gestational age of the population ranged from 30 to 39 weeks, and baseline TSB level ranged from 172 to 390 μmol/L. Regarding the intervention characteristics, the majority of studies utilized LED phototherapy (10/12), and the treatment duration ranged from 14.5 to 68.2 h. A comprehensive summary of the study characteristics was provided in Table 1.

**Table 1:** Summary of the characteristics of the included studies.

Author	Year	Country	Design	Simple size	Gestational week	Mean initial TBS (μmol/L)	Device type	Mean duration (hour)
Bertini et al. (17)	2008	Italy	Cohort	31	30	197	LED	36.4
Eghbalian et al. (21)	2022	Iran	Cohort	108	37	390	Fluorescent	Unknown
El-Farrash et al. (18)	2019	Egypt	RCT	80	38	323	LED	24
Garg et al. (19)	1995	Saudi Arabia	RCT	50	37	290	Fluorescent	24
Karadag et al. (20)	2009	Turkey	RCT	42	38	345	LED	37.9
Kumar et al. (21)	2023	India	Cohort	200	37	352	LED	48
Martins et al. (22)	2007	Brazil	RCT	88	33	172	LED	68.2
Olusanya et al. (23)	2026	Nigeria	RCT	104	39	238	LED	24
Seidman et al. (24)	2000	USA	RCT	69	37	255	LED	31.5
Slusher et al. (25)	2018	USA	RCT	174	38	207	LED	14.5
Sulviani, et al. (26)	2021	Indonesia	Cohort	47	37	331	LED	24
Vandborg et al. (27)	2012	Denmark	RCT	151	36	297	LED	24

RCT, randomized controlled trials; TBS, total serum bilirubin; LED, light-emitting diode.

The methodological quality of the included studies was assessed using the RoB 2 tool for the 8 RCTs (18–20, 22–25, 27) and the NOS checklist for the 4 cohort studies (2, 17, 21, 26). The study “Garg et al. (19)” was rated as “some concerns” in the “Randomization Process” domain due to the lack of clarity regarding the random sequence generation method. The remaining included RCTs were deemed to at low risk of bias (Supplementary Figure S1). The included cohort studies achieved high NOS scores, and no study was excluded (Supplementary Table S1). The reviewers demonstrated substantial agreement during the quality assessment process (Cohen’s *κ* = 0.82).

### 3.3 Intensive phototherapy has greater reduction in TSB levels for neonatal hyperbilirubinemia

**Figure 2:**
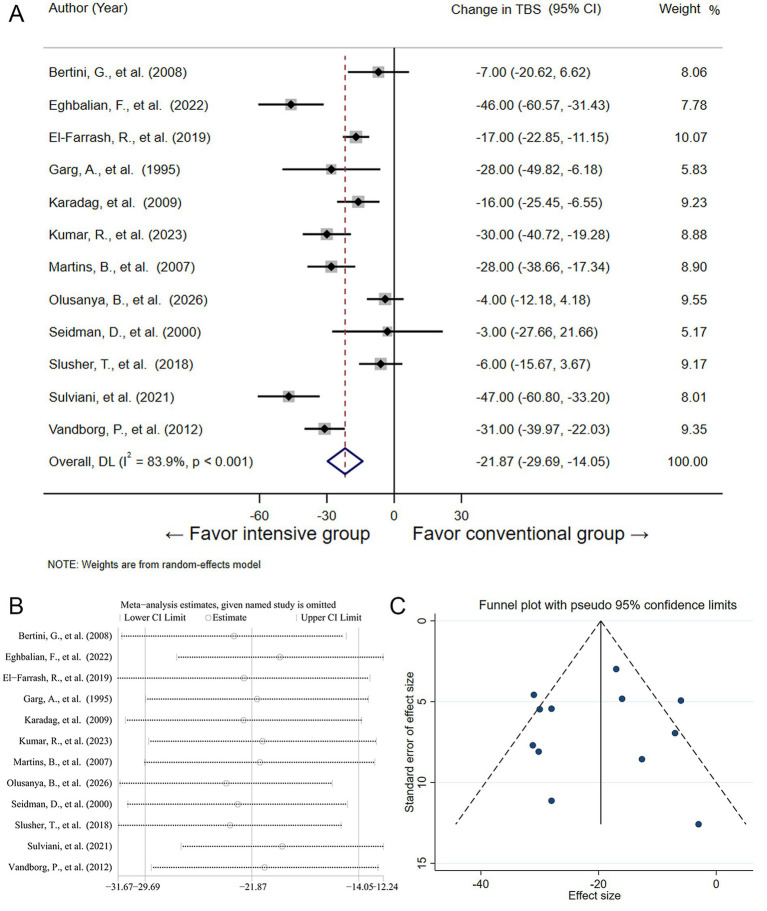
Intensive phototherapy has greater reduction in TSB levels for neonatal hyperbilirubinemia. **(A)** Forest plot of pooled MD for TSB reduction comparing intensive versus conventional phototherapy; **(B)** sensitivity analysis for TSB reduction comparing intensive versus conventional phototherapy; **(C)** funnel plot for assessing publication bias.

A random-effects meta-analysis of 12 studies (2, 17–27) revealed that the intensive phototherapy group achieved markedly greater TSB reductions than the conventional group (MD = −21.87 μmol/L, 95% CI = −29.69 to −14.05, *p* < 0.001). However, the analysis was characterized by substantial heterogeneity (*I*^2^ = 83.9%, *p* < 0.001) (Figure 2A). Influence analysis indicated that no single study disproportionately affected the pooled estimate (Figure 2B). The results of the subgroup analysis showed that the mean gestational age (interaction *p*-value = 0.003) and baseline TSB level (interaction *p*-value = 0.005) were potential source of heterogeneity, whereas study design was not (*p* = 0.106, Supplementary Figure S2A). Mean gestational age ≤37 weeks (MD = −28.75, 95% CI = −37.98 to −19.52) had greater TSB reductions than mean gestational age > 37 weeks (MD = −11.08, 95% CI = −17.98 to −4.19) (Supplementary Figure S2B). In addition, neonates with baseline TSB levels > 257 μmol/L (MD = −29.79, 95% CI = −38.97 to −20.60) achieved greater TSB reductions than those with baseline TSB levels ≤257 μmol/L (MD = −10.27, 95% CI = −20.21 to −0.32) (Supplementary Figure S2C). Furthermore, no significant publication bias (Figure 2C; Begg’s test: *z* = −1.03, *p* = 1.000; Egger’s test: *t* = −0.31, *p* = 0.757) were detected.

### 3.4 Intensive phototherapy significantly reduced the total treatment duration for neonatal hyperbilirubinemia

**Figure 3:**
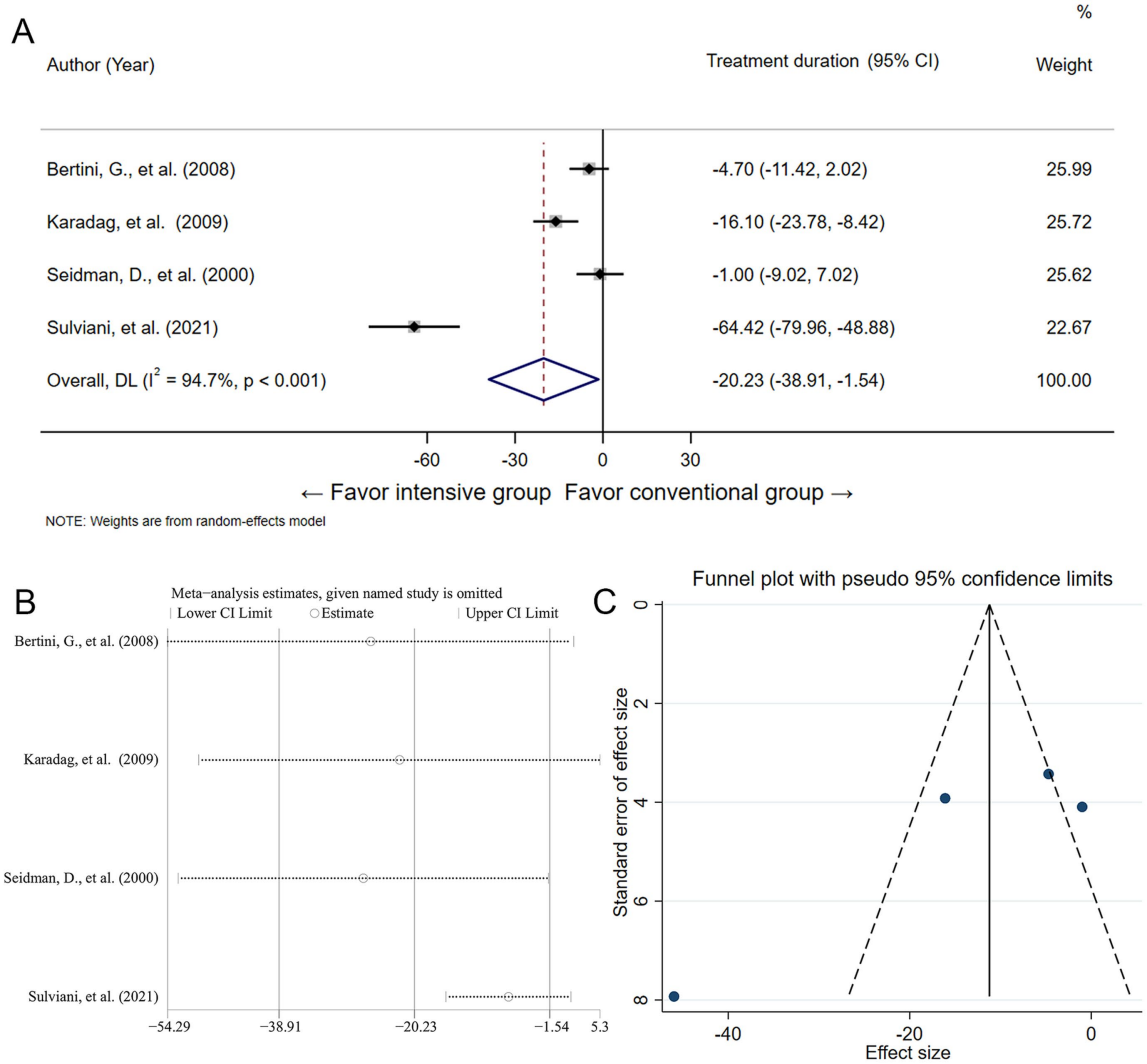
Intensive phototherapy reduced the total treatment duration for neonatal hyperbilirubinemia. **(A)** Forest plot of pooled MD for treatment duration comparing intensive versus conventional phototherapy; **(B)** Sensitivity analysis for treatment duration comparing intensive versus conventional phototherapy; **(C)** Funnel plot for assessing publication bias.

A total of 4 studies (17, 20, 24, 26) were included in the analysis of success rate via random effect model, and the treatment duration from the intensive phototherapy group were shorter than the conventional group (MD = −20.23 h, 95% CI = −38.91 to −1.54, *p* = 0.034). Heterogeneity among these studies was high (*I*^2^ = 94.7%, *p* < 0.001) (Figure 3A). However, no influential publications were detected among the pooled results (Figure 3B). Furthermore, no significant publication bias (Figure 3C) were detected.

## 4 Discussion

Neonatal hyperbilirubinemia poses a substantial risk for permanent central nervous system injury, representing a significant global clinical burden (2). This potential for neurological impairment necessitates timely and effective therapeutic intervention to mitigate long-term sequelae. Phototherapy has been established as the cornerstone of clinical management for unconjugated hyperbilirubinemia and virtually eliminated the need for exchange transfusions, leading to a marked decline in acute bilirubin encephalopathy (4). Mechanistically, phototherapy represents a therapeutic modality that employs light within the 420–480 nm spectrum to reduce TSB levels through photo-oxidation (5). Upon exposure to light at this specific wavelength, bilirubin undergoes isomerization, converting into non-toxic, water-soluble forms that facilitate hepatic metabolism and subsequent systemic clearance (28).

While the spectral characteristics of light are fundamental to the photochemical process, the clinical efficacy of phototherapy is also governed by specific physicochemical parameters of the light source, such as peak emission wavelength, spectral bandwidth, and light irradiance (6). Despite the widespread adoption of phototherapy, the clinical efficacy of intensive versus conventional phototherapy remains a subject of debate. Li et al. (11) meta-analysis found that while home phototherapy reduces parental stress, it is linked to longer treatment duration and higher readmission rates, with very low evidence quality. Crucially, the lack of reported irradiance data prevents a standardized assessment of its efficacy. Our meta-analysis of 12 studies reveals that intensive phototherapy achieves markedly greater TSB reduction compared to conventional phototherapy within the same timeframe. Shortening the duration of phototherapy is a critical neuroprotective strategy. Intensive phototherapy offers comparable efficacy to conventional phototherapy while achieving a significant reduction in treatment duration, hospital length of stay, and overall costs (26). In addition, intensive phototherapy significantly reduced the need for exchange transfusion, proving to be as effective as exchange transfusion itself in lowering TBS levels (29). Supporting this, high-intensity phototherapy demonstrated superior efficacy as a rescue treatment for severe neonatal hyperbilirubinemia with a comparable safety profile, achieving significantly higher success rates and reducing the need for exchange transfusions (30). Collectively, these findings demonstrate that intensive phototherapy represents a promising alternative strategy, offering accelerated bilirubin reduction and minimized treatment duration.

Conventional phototherapy units are constrained by limited irradiance capacity and substantial thermal emission. Conversely, LEDs are characterized by high-intensity narrow-spectrum emission and minimal heat generation and have emerged as viable alternatives. The capacity to position LED units in close proximity to the neonate renders them advantageous devices for the delivery of optimal phototherapy (31). Nizam et al. (12) found that double phototherapy is significantly more effective than single phototherapy in reducing TBS levels, especially in preterm infants and those with birth weights ≥1,500 g. However, ambiguous efficacy across different gestational ages and birth weights limits these conclusions, highlighting the need for more stratified analysis. Crucially, this enhanced efficacy is most pronounced in the preterm neonates with severe hyperbilirubinemia. Subgroup analyses confirm that neonates with a mean gestational age of ≤37 weeks experience significantly greater TSB reductions than their term counterparts. Similarly, neonates presenting with baseline TSB levels exceeding 257 μmol/L achieve markedly superior results compared to those with lower initial levels. Similarly, intensive LED therapy demonstrated a favorable safety profile by significantly reducing the incidence of mild hyperthermia in preterm neonates, alongside fewer side effects and lower operational costs (32). Sherbiny, et al. (30) also demonstrated that high-intensity super LEDs, featuring a narrow wavelength band and near-circumferential coverage, serve as a critical rescue therapy for severe neonatal hyperbilirubinemia. Therefore, the potent bilirubin-lowering capability of high-intensity phototherapy makes it an indispensable and life-saving intervention for managing severe jaundice in vulnerable premature infants.

However, despite these technological advancements, phototherapy may adversely affect the oxidant/antioxidant defense system in hyperbilirubinemic infants, precipitating significant oxidative stress. This process can induce a biochemical imbalance between reactive oxygen species, reactive nitrogen species and endogenous antioxidant levels (18). Hence, clinicians must carefully weigh these benefits against the potential for inducing oxidative stress in vulnerable neonates.

## 5 Limitation

Despite the significant findings of this study, several limitations warrant consideration. First, the efficacy of phototherapy is intrinsically linked to specific treatment protocols; however, the variability in management schemes across different studies precluded standardization, contributing substantially to the high heterogeneity. Second, while the inclusion of older studies offers valuable historical evidence, their generalizability to contemporary clinical practice must be interpreted with caution given the evolution of treatment standards over time. Another limitation of this meta-analysis is the inclusion of both RCTs and observational studies, which vary in methodological quality and susceptibility to bias. This heterogeneity in study design warrants a cautious interpretation of the pooled results. Consequently, there is an urgent need for future large-scale randomized trials that employ strictly standardized protocols.

## 6 Conclusion

Neonatal hyperbilirubinemia poses a significant risk for neurological injury, and phototherapy remains the standard of care. Based on the findings of this meta-analysis, intensive phototherapy appears to be more effective than conventional phototherapy in reducing TSB levels and treatment duration, particularly in preterm infants with severe hyperbilirubinemia. However, considering the moderate certainty of evidence and observed heterogeneity, these results should be interpreted with caution. Future high-quality randomized controlled trials are needed to further validate these findings.

## Data Availability

The original contributions presented in the study are included in the article/Supplementary material, further inquiries can be directed to the corresponding author.

## Supplementary material

SUPPLEMENTARY FIGURE S1 Bias risk assessment for the 8 included RCTs based on the RoB 2 tools.

SUPPLEMENTARY FIGURE S2 Subgroup analyses of TSB reduction comparing intensive versus conventional phototherapy according to (A) study design, mean gestational age (B) and baseline TSB level (C).
