# Supplementary material for: Intensive versus conventional phototherapy for neonatal hyperbilirubinemia: a systematic review and meta-analysis of RCTs and cohort studies
Source: Front Med (Lausanne). 2026 Jun 9;13:1862217. doi: 10.3389/fmed.2026.1862217 (PMC13287029; doi:10.3389/fmed.2026.1862217)
Supplement: SUPPLEMENTARY FIGURE S1 — Bias risk assessment for the 8 included RCTs based on the RoB 2 tools. [file Table_1.docx]

| Table S1. Quality assessment based on the Newcastle-Ottawa Scale (NOS) checklist. | | | | | | | | | | |
| --- | --- | --- | --- | --- | --- | --- | --- | --- | --- | --- |
| **Study** | **Selection** | | | |  | **Comparability** |  | **Outcome** | | **Total score** |
|  | Representativeness of the sample | Sample | Non-respondent | Ascertainment of the exposure |  | Confounding factors are controlled |  | Assessment of outcomes | Statistical test |  |
| Bertini, G., et al. (2008) | * | * | * | * |  | * |  | * | * | 7 |
| Eghbalian, F., et al. (2022) | * | * | * | * |  | ** |  | * | * | 8 |
| Kumar, R., et al. (2023) | * | * | * | * |  | ** |  | * | * | 8 |
| Sulviani, et al. (2021) | * | * | * | * |  | * |  | * | * | 7 |
