# Supplementary material for: Intensive versus conventional phototherapy for neonatal hyperbilirubinemia: a systematic review and meta-analysis of RCTs and cohort studies
Source: Front Med (Lausanne). 2026 Jun 9;13:1862217. doi: 10.3389/fmed.2026.1862217 (PMC13287029; doi:10.3389/fmed.2026.1862217)
Supplement: SUPPLEMENTARY FIGURE S2 — Subgroup analyses of TSB reduction comparing intensive versus conventional phototherapy according to (A) study design, mean gestational age (B) and baseline TSB level (C). [file Table_2.docx]

Table S2. Search strategy of databases

| Databases | Search strategy | Published date | Results |
| --- | --- | --- | --- |
| PubMed | (((((Phototherapy [Title/Abstract])) OR (phototherap* [Title/Abstract])) OR (Light therapy [Title/Abstract])) OR ( Blue light [Title/Abstract])) AND ((((Neonatal Hyperbilirubinemia [Title/Abstract])) OR (Newborn Jaundice [Title/Abstract])) OR (neonat* jaundice [Title/Abstract])) | 1948-2026 | 1183 |
| Web of science | (Phototherapy (Topic) or Phototherap* (Topic) or Light therapy (Topic) or Blue light (Topic)) AND (Neonatal Hyperbilirubinemia (Topic) or Newborn Jaundice (Topic) or Neonat* jaundice (Topic)) | 1900-2026 | 1755 |
| ScienceDirect | （Neonatal Hyperbilirubinemia or Newborn Jaundice） and （Phototherapy or Light therapy or Blue light） | 1997-2026 | 323 |
| EMBASE | ((Neonatal Hyperbilirubinemia or Newborn Jaundice or Neonat* jaundice) and (Phototherapy or Phototherap* or Light therapy or Blue light)).ab. | 1974-2026 | 1360 |
